# Sporadic worldwide “clusters” of feed driven Zilpaterol identifications in racing horses: a review and analysis

**DOI:** 10.1186/s13620-022-00215-8

**Published:** 2022-05-14

**Authors:** Jacob Machin, Kimberly Brewer, Abelardo Morales-Briceno, Clara Fenger, George Maylin, Thomas Tobin

**Affiliations:** 1 Department of Toxicology and Cancer Biology and the Maxwell H. Gluck Equine Research Center, Dept. of Veterinary Science, University of Kentucky, Lexington, KY 40546 USA; 2 15775 Cypress Creek Lane, Wellington, FL 33414 New Zealand; 3 ALSHULA Pharmacy-Veterinary Technical Support, Doha, Qatar; 4 Equine Integrated Medicine, 4904 Ironworks Rd, Georgetown, KY 40324 USA; 5 New York Drug Testing and Research Program, 777 Warren Rd Ithaca, New York, NY 14853 USA

**Keywords:** Horses, Feed, Zilpaterol, Inadvertent transfer, Urine, Limit of Detection (LOD), Worldwide clusters

## Abstract

Zilpaterol is a *β2-*adrenergic agonist medication approved in certain countries as a cattle feed additive to improve carcass quality. Trace amounts of Zilpaterol can transfer to horse feed, yielding equine urinary “identifications” of Zilpaterol. These “identifications” occur because Zilpaterol is highly bioavailable in horses, resistant to biotransformation and excreted as unchanged Zilpaterol in urine, where it has a 5 day or so terminal half-life.

In horses, urinary steady-state concentrations are reached 25 days (5 half-lives) after exposure to contaminated feed. Zilpaterol readily presents in horse urine, yielding clusters of feed related Zilpaterol identifications in racehorses. The first cluster, April 2013, involved 48 racehorses in California; the second cluster, July 2013, involved 15 to 80 racehorses in Hong Kong. The third cluster, March 2019, involved 24 racehorses in Mauritius; this cluster traced to South African feedstuffs, triggering an alert concerning possible Zilpaterol feed contamination in South African racing. The fourth cluster, September/October 2020 involved 18 or so identifications in French racing, reported by the French Laboratories des Courses Hippiques, (LCH), and in July 2021, a fifth cluster of 10 Zilpaterol identifications in South Africa.

The regulatory approach to these identifications has been to alert horsemen and feed companies and penalties against horsemen are generally not implemented. Additionally, given their minimal exposure to Zilpaterol, there is little likelihood of Zilpaterol effects on racing performance or adverse health effects for exposed horses.

The driving factor in these events is that Zilpaterol is dissolved in molasses for incorporation into cattle feed. Inadvertent incorporation of Zilpaterol containing molasses into horse feed was the source of the California and Hong Kong Zilpaterol identifications. A second factor in the 2019 Mauritius and 2020 French identifications was the sensitivity of testing for Zilpaterol in Mauritius and France, with the French laboratory reportedly testing at a “*more sensitive level for Zilpaterol*”. As of January 1^st^, 2021, the new FEI Atypical Finding (ATF) policy specifies Zilpaterol as a substance to be treated as an Atypical Finding (ATF), allowing consideration of inadvertent feed contamination in the regulatory evaluation of Zilpaterol identifications.

## Background

**Fig. 1:**
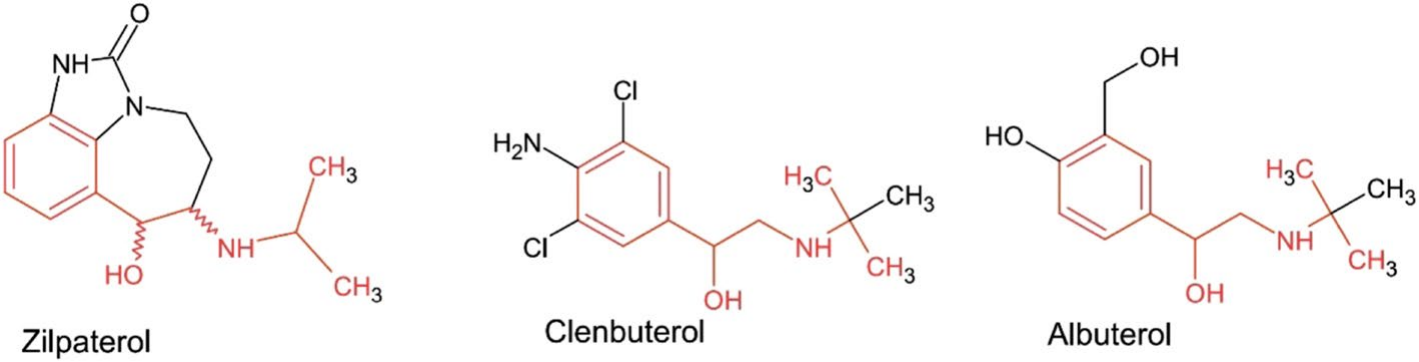
Zilpaterol, Clenbuterol and Albuterol, structurally related β2-agonists: Zilpaterol, ( ±)-*trans*-4,5,6,7-Tetrahydro-7-hydroxy-6-(isopropylamino)-imidazo[4,5,1-jk]-[1]benzazepin-2(1*H*)-one, formula, C_14_H_19_N_3_O_2,_ molar mass 261.325 g/mol. Zilpaterol contains two chiral carbons, carbons 6 and 7, giving rise to four enantiometic forms of Zilpaterol, (6R,7R), (6R,7S), (6S,7R). (6S,7S). Zilpaterol is marketed as Zilmax [3], a racemic mixture of the pharmacologically active 6R,7R eutomeric *β2*-agonist isomer and also the 6S,7S enantiomer.

Zilpaterol, ( ±)-*trans*-4,5,6,7-Tetrahydro-7-ydroxy-6-(isopropylamino)-imidazo[4,5,1-jk]-[1]benzazepin-2(1*H*)-one, C_14_H_19_N_3_O_2,_ molar mass, 261.325, Fig. 1 below, is a β2-adrenergic agonist approved in a number of countries as a cattle feed additive, where it promotes weight gain and improves carcass quality [1]. The recent reporting of 18 or so feedstuff related low concentration identifications of Zilpaterol in urine samples from racing horses by the French racing laboratory, Laboratory des Courses Hippiques (LCH) [2] and previous clusters of Zilpaterol identifications elsewhere (Table 1, below) have given rise to questions related to the origins of such identifications, the possibility or otherwise of performance effects on racing horses and also any short or long term equine health consequences related to such low concentration exposures.

**Table 1:** Feed related clusters of equine zilpaterol identifications

Year	Country	Horses	Detection	Control Authority	Lab	Horse Feed	Penalty
March April 2013	USA California	48	Post-Race	California CHRB	University of California, Davis	1- US Brands	No Trainer Penalties
June 2013	Hong Kong	80 plus	Pre-Race	Hong Kong Jockey Club (HJKC)	HKJC Lab	2 US Brands	No Trainer Penalties
2019	Mauritius	24	Out of Competition	Mauritius Turf Club	QuantiLAB	South African	No Trainer Penalties
2020	France	18	Post-Race	France Galop, Le Trot	LCH France	Irish	No Trainer Penalties
July 2021	Republic of South Africa	10	Post-Race	National Horse- racing Authority (NHA)	National Horse-racing Authority	South African	1 Trainer guilty, 7 trainers retained counsel

Feedstuff related clusters of Zilpaterol identifications, 2013→2021, Dates of events, Jurisdiction, Number of cases reported, Pattern of testing, Authority, Laboratory, Source of Feed and Penalties. In Hong Kong 16 horses were initially identified but there were suggestions that at least 80 more horses had been exposed. In the 2020 events 18 horses were withdrawn from racing in England and France because of exposure to Zilpaterol containing horse feed

In equine forensic science a “cluster” is defined as at least three identifications of the same substance in the same geographic area and time frame in horses from three or more independent trainers [4]. This definition is based on the unlikelihood of three unrelated trainers independently and simultaneously deciding to use the same inappropriate substance in their racing horses. These reviewed Zilpaterol identifications readily meet this definition, in that these clusters involve relatively large numbers of unrelated horses/trainers in defined locations and time frames. The concentrations of the substance involved in such clusters are also likely to be pharmacologically irrelevant, a further indication that these trace level identifications of Zilpaterol are not trainer associated, as is the case in these current matters.

In the absence of defined regulatory “cut-offs” for the detection of Zilpaterol, horsemen competing under these regulatory conditions require guidance for determining the time post-withdrawal of affected feed for a horse testing “positive” for Zilpaterol to go analytically “negative” [5, 6]. Addressing these concerns, we have reviewed the available data on urinary concentrations of Zilpaterol in horses and other animals in order to determine the pharmacological and toxicological significance of trace level identifications of Zilpaterol in equine urines and the time required for such horses/urines to go analytically “negative”. We will begin this analysis with a review of the chemical structure of Zilpaterol and its pharmacology and pharmacokinetics in horses and other species.

### The unique chemical structure of Zilpaterol 

Zilpaterol was synthesized for use as an oral β2-agonist feed additive administered to cattle over periods of several weeks [1]. It is therefore useful if the molecule is 1/ well absorbed orally and, 2/ has a long plasma half-life, i.e., remains present as the pharmacologically active substance in the blood stream of treated animals. Zilpaterol meets both of these requirements, making it a useful partitioning agent and it is currently approved for such use in cattle in the US, Canada, Brazil, Colombia, Costa Rica, Dominican Republic, Guatemala, Honduras, Kazakhstan, Mexico, Nicaragua, Panama, Peru, South Africa, South Korea, and the Ukraine [1, 3].

### Pharmacokinetics and pharmacodynamics of Zilpaterol

Zilpaterol, Fig. 1 above, meets these pharmacological requirements by virtue of its chemical structure. Zilpaterol is unique among marketed *β2*-agonists in that the *β2-*agonist portions of the Zilpaterol molecule are held in a specific configuration by the tricyclic ring structure of Zilpaterol, [7] in comparison with the less constrained structures of Clenbuterol and Albuterol, both of which are more rapidly biotransformed [8, 9]. This chemical structure apparently accounts for Zilpaterol’s rapid and approaching 100% oral bioavailability [10] and its resistance to metabolic biotransformation, which results in a prolonged 5 day or so terminal half-life of Zilpaterol in equine urine, a pharmacokinetic fact that has important regulatory implications.

The regulatory implications are that pharmacodynamically Zilpaterol presents as a classic *β2*-agonist partitioning agent with a considered ability to improve muscle mass and thereby potentially enhance athletic performance or yield a competitive advantage. This analysis has led to a number of regulatory organizations to ban Zilpaterol completely in animals and humans (ARCI 2018, WADA, 2018). As a practical matter, this meant that prior to January 2021 any detection of Zilpaterol in a racing sample initiated regulatory review under a zero tolerance Zilpaterol detection policy, including detection of low picogram/ml concentrations of Zilpaterol in post-race urine samples.

### Urinary pharmacokinetics Zilpaterol

The best available data on post administration equine urinary concentrations of Zilpaterol are those of Shelver and colleagues [10–13]. In their equine experiments Zilpaterol was administered at the recommended food animal dose of 0.17 mg/kg of body weight, for a total daily dose of about 70 mg per horse to three horses weighing about 470 kg. Because these dosed horses immediately showed adverse clinical responses [10, 14], namely increased heart rate, tremors and profuse sweating, the dose was reduced substantially for day 2 in these horses. These experimental data are therefore based on this two dose administration, resulting in high initial urinary concentrations of Zilpaterol, on the order of 10,000 nanograms per ml in urine. These findings are consistent with rapid and complete absorption of Zilpaterol as well as the adverse responses seen in these experimental horses. The data from Shelver et al. are consistent with a two-compartment Zilpaterol urinary elimination model, as in equation #1 below:

1 $$8967.{436e}^{-1.32202t}+{13.86484e}^{-0.13597*t}$$

**Fig. 2:**
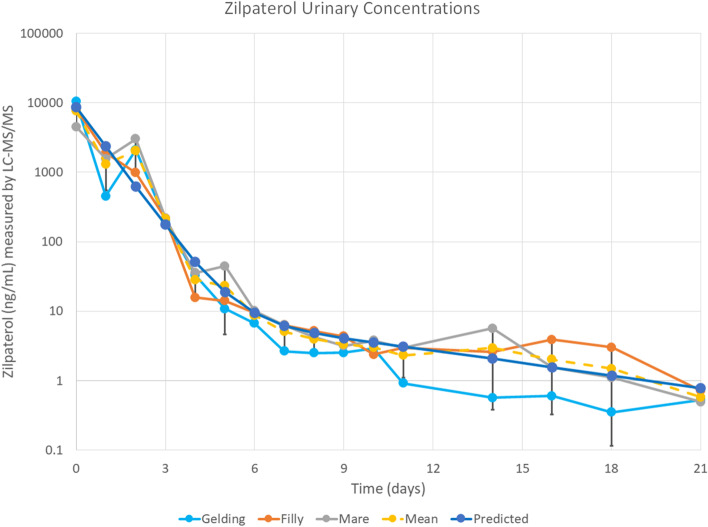
Mean urinary concentrations of Zilpaterol post 0.17 mg/kg (~ 70 mg/horse) PO., replotted from Shelver et al [10]. The individual horses are represented by the teal, orange, and grey lines, and symbols. Mean values of the three horse data set are represented by the yellow circles and dashed line, with standard deviation of the groups at each timepoint represented by the black bars. The dark blue circles represent our pharmacokinetic model, calculated by curve stripping of the mean dataset to determine a multi-exponential decay model showing biphasic elimination. The first phase has a calculated half-life of 13.2 h, while the much slower terminal phase has a calculated half-life of 120.7 h, with the transition between these domains at approximately 6 days post treatment

This pharmacokinetic model was calculated by curve stripping of the mean data set and describes a multi−exponential decay model showing biphasic elimination kinetics as described by Dunne et al., [15]. The initial rapid phase shows a urinary elimination half−life of about 13.2 h, followed by a second much slower and apparently terminal urinary elimination half−life of around 120.7 h, or close to 5 days, as in Fig. 2 below.

The initial urinary half-life of about 13.28 h is observed for the first 5 days post administration. Then, starting about day 6 post administration, the slower and presumably terminal phase urinary half-life presents, with an apparent terminal half-life of about 121 h or close to 5 days. In these experiments Zilpaterol remained detectable in urine at about the one nanogram per milliliter Limit of Detection (LOD) of the analytical methodology for the full 21 days of this experiment. We also note that this 1 nanogram/ml urinary concentration is a full 10,000 fold below the peak plasma concentrations achieved in these horses and which plasma concentrations produced clear pharmacological responses.

Consistent with the observed adverse clinical responses in these horses, the initial urinary concentrations of Zilpaterol were estimated at about 10 times the concentrations reported in bovines and sheep [11–13] and 100 times the concentrations reported in pigs [13]. The basis of the pharmacokinetic differences between these species is unclear, but it is apparent that administered as a one-time oral dose, Zilpaterol is rapidly and completely absorbed by the horse, yields higher than expected plasma concentrations as judged by the adverse clinical responses reported, and is also excreted initially at relatively high concentrations in equine urine [10, 14]. These data show that for reasons which presumably relate to the anatomical and physiological differences between the equine and ruminant digestive tracts, that the equine foregut rapidly and essentially completely absorbs Zilpaterol, in contrast with its initial dilution in the rumen of cattle and sheep. The take home message at this point is that in the horse Zilpaterol administered orally is rapidly and completely absorbed.

The second take home message from these data is that the terminal urinary half-life of Zilpaterol in the horse is relatively prolonged [10], at five days or so, which has important implications for the urinary detection of Zilpaterol following daily exposure of horses to trace amounts in feed, as we will now detail.

### Zilpaterol exposure and urinary Zilpaterol concentrations

The Shelver equine studies [10–13] demonstrate the pharmacokinetic profile of Zilpaterol after a two dose regimen, which is unlikely to reflect the exposure of horses in our described “clusters” of urinary identifications resulting from ongoing trace level dietary exposure. To our knowledge the only available data on ongoing trace level dietary exposure in any animal are those of Smith et al., 2019, who studied the relationship between dietary exposure to trace amounts of Zilpaterol and urinary concentrations in sheep [12]. In these experiments, sheep were administered Zilpaterol in feed at total daily doses of 13 µg, 130 µg and 1,300 µg or 1.3 mg/day/sheep for 12 days. The mean weight of these sheep was fractionally above 50 kg/sheep, or about 10% of the weight of an adult horse. Each of these daily administration protocols resulted in readily detectable peak urinary concentrations of Zilpaterol, reported as 2.8 ng/ml for the low dose, 21.4 ng/ml for the medium dose and 218 ng/ml for the high dose, these urinary concentrations relating directly to the daily dose of Zilpaterol, as specifically noted by Smith and his colleagues. Given the approximately tenfold body mass difference between a sheep and a horse, exposure to the low 13 mcg daily dose administered to these sheep may be expected to yield 280 pg/ml urinary Zilpaterol concentration in a horse, assuming that sheep and horses handle Zilpaterol broadly similarly, and which urinary concentrations are readily detectable by modern racing chemistry laboratories.

### The forensic significance of the longer terminal urinary half-life of Zilpaterol

Horses exposed to Zilpaterol in feed are likely to present steady state urinary concentrations of Zilpaterol, because contaminated feed produced and purchased in batches is likely to be consumed over the course of many days. Maximal steady state urinary concentrations of Zilpaterol will be achieved after five urinary half-lives or about 25 days of daily exposure to Zilpaterol. Because Zilpaterol is completely absorbed orally, is not significantly metabolized, and is slowly eliminated by the horse, urinary concentrations of Zilpaterol will increase post exposure, peaking at this 25 day mark. By day 25 or so the amount of Zilpaterol eliminated each day is equivalent to the amount being absorbed and will be the highest urinary concentration of Zilpaterol associated with that particular dose / feed exposure level. Then, when exposure to the Zilpaterol containing feedstuff ceases, the urinary concentrations of Zilpaterol will decline, following this same characteristic 5 day or so terminal urinary half-life.

### Significance of overall Zilpaterol pharmacokinetics

These equine Zilpaterol pharmacokinetics immediately explain the events underlying the Table 1 reported clusters of trace level Zilpaterol identifications following exposure of horses to trace amounts of Zilpaterol in equine feedstuffs. First, Zilpaterol is highly bioavailable, one of the few substances listed as 100% orally bioavailable, consistent with the rapid and complete absorption of orally administered Zilpaterol in the Shelver horse experiments [10]. Second, and unusually, Zilpaterol is not significantly metabolized by the horse, also consistent with the high initial urinary concentrations of Zilpaterol post administration. This resistance to metabolism is also consistent with the third unusual aspect of Zilpaterol in horses, namely the relatively long terminal plasma half-life of Zilpaterol in the horse. Together, these pharmacokinetic characteristics give rise to the ongoing feed related trace level exposure to Zilpaterol and resulting “cluster” identifications across multiple racing jurisdictions worldwide.

### The time required for a Zilpaterol “positive” urine to go “negative”

Important to horsemen competing in regulatory environments in the absence of a regulatory threshold to control environmental-source Zilpaterol is the time required after Zilpaterol exposure ceases for a Zilpaterol “positive” urine to go analytically “negative”. The answer to this question is variable since it depends on, 1) the concentration of Zilpaterol in the horses’ urine at the time that exposure to Zilpaterol ceases and 2) the sensitivity, technically the Limit of Detection [LOD] of the analytical method in use by the testing laboratory [5, 6]. In the absence of information as to the concentration present in the urine sample at the time of identification of the problem and the fact that equine drug testing laboratories are usually reluctant to share their in place Limits of Detection [LOD] for substances such as Zilpaterol, withdrawal time estimates may be little more than educated estimates. Furthermore, as per the Irish Horseracing Regulatory Board (IHRB), October 16^th^, 2020, communication, [2, 16] some laboratories may be “*operating to a more sensitive level for Zilpaterol” *[2] than others, adding to the uncertainly associated with such estimates. In this regard, the most practical option is elective testing, which allows one to determine whether or not the sample in question is Zilpaterol *“positive”* or *“negative”* as per the testing technology of the laboratory performing the analysis.

### The role of testing sensitivity in these matters

It is an analytical fact of life that for a substance such as Zilpaterol equine drug testing laboratories do not all function at the same level of testing sensitivity. With respect to these clusters of Zilpaterol identifications, this differential in testing sensitivity first became apparent in association with the 2019 cluster of Zilpaterol identifications in Mauritius [17]. This is because the sensitive testing in place in the Mauritius Laboratory, QuantiLAB Ltd, was detecting urinary Zilpaterols traced to horse feeds originating in South Africa [18]. This identification by the Mauritius laboratory of Zilpaterol in South African horse feeds apparently led to a communication to horsemen by the South African National Horseracing Authority (NHA) concerning the possible presence of Zilpaterol in South African horse feeds. Additionally, to our knowledge as of July 2021 the first Zilpaterol “positives” have been called in South African racing, with 10 Zilpaterol identifications from 8 trainers recently reported in South African racing [19].

A similar inter-laboratory differential in Zilpaterol testing sensitivity is apparent in the recent French 2020 cluster of Zilpaterol identifications, where Zilpaterol identifications in regulatory samples were reported only by the French LCH laboratory, and not by the English LGC laboratory analyzing British and Irish racing and Fédération Èquestre Internationale (FEI) samples. Reportedly, as in Table 2 above, the LGC laboratory does not report Zilpaterol at urinary concentrations of less than 250 picograms/ml, while the French LCH laboratory reports Zilpaterol down to 100 picograms/ml, a 2.5 times more sensitive test. This more sensitive testing by the LCH laboratory presumably accounts for the fact that horses consuming potentially Zilpaterol containing feeds have not been reported as producing Zilpaterol containing urines in English and Irish equine drug testing, while there have been a number of such identifications reported in French regulatory testing (Table 1). Other fallout from these 2020 French events included 11 horses being withdrawn from the 2020 Prix de l'Arc de Triomphe in October 2020 [20], and a trainer in England withdrawing 7 runners, all associated with exposure or potential exposure to Zilpaterol contaminated feed.

**Table 2:** Laboratories associated with the french 2020 Zilpaterol cluster

Lab Name	Lab ISO 17025 Accredited	Method Used Is LC–MS-MS in Scope	Matrix in Scope	Limit of Detection
LGC- Newmarket, UK and IHRB Analytical Laboratory Also reference lab on France Gallop list of Approved Labs	Yes	Yes	Water Liver Urine	1 ppb 1 ppb 0.25 ppb
Irish Equine Centre, Co. Kildare	Yes	Yes	Urine ( Bovine)	1 ppb
DAFM, Backweston, Co Kildare	Yes	Yes	Feed	50 ppb
Independent Equine Nutrition Mildenhall, UK Dr. Mark Dunnett	No	No	Feed	1–10 ppb as conducted negative controls on samples
LCH Verrieres-le-Buisson, France	Yes	Yes	Urine	0.1 ppb (as reported)
NHA Laboratory	Yes	Yes	Urine	Not Reported

Testing laboratories involved in the 2020 French Zilpaterol Cluster and best available estimates of Zilpaterol testing sensitivities linked to the 2020 Zilpaterol cluster, sensitivity reported as parts per billion (ppb) in urine. Irish/British racing samples were tested at the LGC laboratory and the French racing/equine samples were tested at Laboratories des Courses Hippiques, (LCH). Samples were also tested at the Irish Equine Center (IEC) laboratory, the Backweston laboratory of the Irish Department of Agriculture, Food, and the Marine (DAFM), and Independent Equine Nutrition (IEN) Laboratory, Mildenhall, UK. Best available information is that the Limit of Detection for LCH Zilpaterol urine testing was in the order of 100 pg/ml, 2.5 times more sensitive than that of LGC Newmarket

### These trace level Zilpaterol identifications are most likely without pharmacological or toxicological significance for the horses involved

The final question with regard to these clusters of Zilpaterol identifications is their significance for the health, welfare, and racing performance of the involved horses. The answer to this question is clear; given the low daily mcg amounts of Zilpaterol to which these horses have been exposed, in the order of 1/4,000 or less than a pharmacologically effective dose, the likelihood of significant adverse health effects, either short or long term is, as a practical matter, indistinguishable from zero. In this regard the European Union Joint Expert Committee on Food Additives [JECFA] [21] derived an acceptable daily intake of Zilpaterol for humans of 0.04 µg per kilogram body weight, or a total daily dose for human of about 2.8 µg per day, or about 17 ug/day for a horse [6]. These dosage levels, based on acceptable human exposure rates, are on the same order of the15 ug/day or so total dose of Zilpaterol to which the horses in the most recent 2020 French cluster of racing chemistry Zilpaterol “positives” have been exposed. There is therefore as a practical matter, essentially no likelihood of adverse effects on these animals related to the Zilpaterol exposure giving rise to these low concentration French racing chemistry “positive” urinary identifications. Similarly, there is also no significant possibility of an effect on the racing performance of horses exposed to these extremely small amounts of Zilpaterol.

### Cluster events case reports

These data and analyses show that Zilpaterol has chemical and pharmacokinetic characteristics that result in its presence at detectable concentrations in equine urine samples even though the horses have only been exposed to daily microgram amounts of Zilpaterol. In these situations, dietary exposure to small daily intakes of Zilpaterol can give rise, over a matter of 2–4 weeks, to racetrack testing detectable urinary concentration of Zilpaterol, i.e., potential Zilpaterol “positives’, as we will now detail [2].

#### California, 2013

The first well characterized Zilpaterol identification cluster took place in March / April 2013 in California Racing, where a number of US feed products from a California plant inadvertently came to contain small amounts of Zilpaterol. This Zilpaterol began to show up in racing horses, apparently about two weeks after their first exposure to the affected feed, consistent with the urinary accumulation time course set forth above. A total of 48 horses were reported “positive” for Zilpaterol before the source was identified and the problem remedied. The California racing authorities also recognized that the horsemen involved were entirely innocent and our understanding is that no regulatory action was taken against the affected horsemen [22, 23].

#### Hong Kong, 2013

Soon thereafter, in July 2013, a similar Zilpaterol identification cluster unfolded in Hong Kong, where at least 16 horses were reported as testing positive for Zilpaterol. The source of Zilpaterol was again traced to feed and since the horses were racing at Hong Kong Jockey Club (HKJC) tracks, the feed had been provided via the Hong Kong Jockey Club itself. The total number of Zilpaterol identifications reported in this Hong Kong cluster was 16, although there have been suggestions that a larger number, possibly 80 or so other horses racing at Hong Kong, may have been exposed. Additionally, and quite interestingly, we understand that the feed products causing these Hong Kong identifications had become contaminated from the same Zilpaterol source as the earlier California identifications, with the time delay between the California and Hong Kong identifications reflecting transpacific shipping time [24, 25].

#### Mauritius, 2019

The next cluster of Zilpaterol identifications was in March 2019, when the racing stewards of the Mauritius Turf Club reported that Zilpaterol had been detected in Out Of Competition (OOC) urine samples taken from 24 horses from 7 different stables. The Stewards considered that it was beyond reasonable doubt that the horses had tested positive for Zilpaterol as a result of feed contamination, so no action was taken against the affected trainers. In a later communication, dated November 25^th^, 2019, the Mauritius Turf Club authorities reported that an investigation had concluded that the most likely source of these Zilpaterol identifications was feed originating in South Africa [26, 27].

Shortly before the Mauritius Turf Club authorities released their November 25th, 2019 report, concerns about possible Zilpaterol detections in racing horses in South Africa were communicated, although to our knowledge no formal “positive” identifications were “called”. On November 8^th^ 2019 the South African Sporting Post noted that the National Horseracing Authority [NHA] in South Africa, in an unsigned and undated notice, stated that “*in some racehorse specimens emanating countrywide, traces of a substance which may be indicative of Zilpaterol”,* effectively communicating the possible presence / detection of Zilpaterol in South African racing samples [16]. This communication was greeted with concern by South African trainers, who were unclear as to what the South African NHA expected the trainers to do, besides contacting their feed merchants and requesting confirmation, which may well have been the reason for the NHA communication [18, 28, 29]. We also note that these Zilpaterol concerns in South African racing are fully consistent with approval of Zilpaterol for use in cattle feed in South Africa and the now well understood ease with which Zilpaterol can inadvertently transfer in microgram amounts from livestock industry sources to horse feed and thereby to post-race urine samples.

#### France 2020

The 2020 cluster of Zilpaterol racing identifications occurred in France, involving 12 France Galop Thoroughbred horses, 4 Le Trot Harness Horses and 2 horses from trainer’s yards, we understand to date an 18 total samples, with the testing performed by the French Laboratory des Course Hippiques (LCH) [30, 31]. The first samples in which identifications were reported were collected on or about August 30^th^, with the first identifications of Zilpaterol reported by LCH on September 29th. The apparent source of Zilpaterol in these samples has been microgram amounts of Zilpaterol inadvertently incorporated into some horse feed products, the estimated daily intake per horse being minimal, in the order of 15 ug/horse/day. This French Zilpaterol cluster is unusual in that the identifications were reported only in France and are associated only with LCH testing, even though the feeds in question were also consumed by horses racing in England and Ireland [32, 33]. This French Zilpaterol cluster is therefore forensically similar to the 2019 Mauritius cluster, where the Mauritius Zilpaterol identifications were traced to South African horse feeds, but these feeds had apparently not drawn attention in South African racing until the more analytically sensitive Mauritius identifications were traced back to a South African horse feed source.

#### South Africa, 2021

As this report was being drafted the National Horseracing Authority in South Africa has apparently elected to call 10 Zilpaterol “positives” on 8 trainers in the KwaZulu-Natal province [19], apparently another classic “cluster”. Seven of these trainers have retained counsel and one trainer with two “positives” pleaded “not guilty”. Despite the extensive worldwide precedents set forth above, this trainer was found “guilty” by the NHA and issued a warning. The downside for this trainer is that he now has an Association of Racing Commissioners International (ARCI) Drug Class 2, Penalty Class A foreign substance violation on his record, despite being to our knowledge no more guilty of such a violation than any of the other 100 or so trainers worldwide with similar feed related trace level Zilpaterol “positives”.

### Regulatory approaches to these Zilpaterol clusters

Reviewing these 2020 Zilpaterol cluster events, the International Federation of Horseracing Authorities (IFHA) and the European Horseracing Scientific Liaison Committee, (EHSLC) have recommended that all horseracing jurisdictions should offer elective testing for Zilpaterol where feed contamination is suspected. This elective testing should be performed in the country in which the horse holds an entry, and no regulatory action should be taken against any screening findings for Zilpaterol in an elective test where it can be demonstrated that the horse was likely fed contaminated feed [33]. The final recommendation was that working through the IFHA, racing jurisdictions and their analytical laboratories will work together to harmonize the reporting limits for Zilpaterol and other key substances which are prohibited at all times.

With respect to the regulatory significance of these clusters of Zilpaterol identifications, it is important to note that to our knowledge no significant regulatory actions have been taken against any of the horsemen involved. [34] In the 2103 Hong Kong cases, triggered by postrace identifications, the Hong Kong Jockey Club stewards conceded the Zilpaterol finding was due to the “*feed product imported by the Club at the request of the trainers,” “being contaminated”* [35]*.* Given this circumstance, the trainer involved was not penalized, because the stewards considered that the Hong Kong trainers involved were innocent of any wrongdoing in these Hong Kong Zilpaterol matters, although the Zilpaterol positive horses were disqualified.

The concentrations of Zilpaterol involved in these identifications are considered to be without pharmacological or forensic significance, and at times the amounts detected are defined simply by the limit of sensitivity of the testing in place in the testing laboratory question, as in the 2019 Mauritius and 2020 French clusters. In the Mauritius matter, the Mauritius Turf Club reported on March 22^nd^, 2019, that “*after due consideration, the Racing Stewards found, beyond reasonable doubt, that the above horses had been tested positive for ‘Zilpaterol’ as a result of feed contamination.* [36]*. Accordingly, no action was taken against any one of the above trainers”*. Similarly, as reported by the Blood-Horse on March 27^th^, 2013, “*the California Horse Racing Board, citing feed contamination, has dismissed all 48 positive tests for Zilpaterol”.* [37].

Consistent with these rulings, the British Equestrian Federation (BEF) on or about October 24^th^, 2020, instituted “*a 14 day moratorium over positive doping tests”.* During this moratorium any horse returning a positive result for Zilpaterol will not be subjected to any regulatory action under the BEF anti-doping rules, provided the positive sample is consistent with the feed being contaminated with Zilpaterol [38]. This moratorium was to be kept under review and was described as “*may be extended depended depending upon updating information relating to the contamination*. Additionally, the British Equestrian Trade Association (BETA) indicated that there were “*no health or welfare issues in a horse consuming feed containing the level of Zilpaterol found.* [36].

In a further response to these events, on the 23rd of November 2020, the Fédération Èquestre Internationale[FEI] proposed a new analytical finding category called an Atypical Finding (ATF) [39]. We note that these Zilpaterol identifications meet each of the ATF policy criteria recently presented by the FEI. As set forth in this policy, when reviewing a potential Adverse Analytical Finding [AAF] for consideration as an Atypical Finding [ATF], the FEI will take a number of factors into account, all of which factors are, to our knowledge, met by the Zilpaterol identifications reviewed in this communication.

These FEI ATF policy factors/criteria include a requirement that there be identifications of the same prohibited substance arising from other samples taken at the relevant event(s), a criterion met by the various Zilpaterol identifications clusters reported in this communication. The second criterion is that there be ATF's arising from the same prohibited substance from other samples taken in previous events held at the same venue and or in the same region, which criterion is also very obviously met. The third criterion is that samples taken from feed or bedding at the relevant event test positive for the substance in question, which criterion has also been met by these Zilpaterol identifications. The FEI also addresses the matter of the concentration of the particular prohibited substance in the analytical samples which criterion is to our knowledge also met in these Zilpaterol matters. Finally, the new FEI Atypical Findings policy specifies Zilpaterol as a prohibited substance that will be treated as an Atypical Finding as of January 1^st^, 2021.

## Conclusions

In closing, it appears that these horse racing Zilpaterol cluster events have been driven in large part by a worldwide cattle feed manufacturing process. During cattle feed manufacturing, Zilpaterol is incorporated into the feed by dissolving it in the liquid molasses component of the feed product. Inadvertent incorporation of Zilpaterol containing molasses into horse feed has apparently been the driving factor in each of these horse racing related clusters of Zilpaterol identifications.

In the California horse racing identifications in the spring of 2013 the source of the Zilpaterol contamination was identified as a Zilpaterol containing molasses product intended for bovine use that inadvertently came to contaminate the feedstuffs products involved in this California matter. Then, at the same time as these California Zilpaterol identifications were being evaluated, shipments of horse feeds similarly contaminated with Zilpaterol containing molasses were in transit to Hong Kong. These products arrived in Hong Kong a number of weeks after the California sequence of Zilpaterol events and three months later, gave rise to the sequence in Hong Kong of Zilpaterol identifications.

The 2019 Mauritius cluster of Zilpaterol identifications were linked to South African feedstuffs, and the 2020 French identifications of Zilpaterol were also linked to South Africa. Reviewing the 2020 French cluster, the Irish Department of Agriculture, Food and the Marine (DAFM) noted that *“The contamination was traced back to a sugar mill in South Africa that operates with only one blend tank at its feed site. This tank is used for blending standardized molasses, containing zilpaterol, for the local South African market. The blend tank was not sufficiently cleaned for use of production of sugar cane molasses for export and this resulted in a cross-contamination with zilpaterol of sugar cane molasses exported to Ireland. The necessary measures have been put in place to prevent/avoid re-occurrence of such a cross-contamination”* [40]*.* This South African shipment of molasses containing Zilpaterol also went to a significant number of English horse feed companies as reported by the British Equestrian Trade Association [41], although as noted, only horses subjected to the higher sensitivity LCH French testing for Zilpaterol were identified as regulatory “positive” identifications. South Africa is one of the countries in which the use of Zilpaterol as a bovine feed additive has long been approved, consistent with horse feed from South Africa also being identified as the source of the 2019 Mauritius Zilpaterol cluster and presumably also the most recent 2021 cluster of Zilpaterol identifications in South Africa.

## Data Availability

The datasets used and/or analyzed during the current study are available in the public domain as referenced in the manuscript or from the corresponding author on reasonable request.

## Abbreviations

LCH: Laboratories des Courses Hippiques (France)
LOD: Limit Of Detection
CHRB: California Horse Racing Board
HKJC: Hong Kong Jockey Club
NHA: National Horseracing Authority ( South Africa)
US: United States ( of America)
IHRB: Irish Horseracing Regulatory Board
UK: United Kingdom
LGC: Laboratory of the Government Chemist (Fordham, England)
FEI: Fédération Èquestre Internationale
JECFA: Joint Expert Committee on Food Additives [European Union]
OOC: Out Of Competition
EHSLC: European Horseracing Scientific Liaison Committee
ARCI: Association of Racing Commissioners International
IFHA: International Federation of Horseracing Authorities
BEF: British Equestrian Federation
BETA: British Equestrian Trade Association
ATF: Atypical Findings
DAFM: Department of Agriculture, Food, and the Marine (Irish)
